# In vitro protein digestibility and biochemical characteristics of soaked, boiled and fermented soybeans

**DOI:** 10.1038/s41598-021-93451-x

**Published:** 2021-07-09

**Authors:** Sunantha Ketnawa, Yukiharu Ogawa

**Affiliations:** 1 Graduate School of Horticulture, Chiba University, Matsudo, Chiba 271-8510 Japan; 2 Department of Biochemistry, Faculty of Medicine, Chiang Mai University, Chiang Mai, 50200 Thailand; 3 Functional Food Research Center for Well-Being, Chiang Mai University, Chiang Mai, 50200 Thailand

**Keywords:** Biochemistry, Plant sciences, Health care, Nutrition

## Abstract

Protein digestibility of soybean obtained from the main manufacturing steps for natto, such as soaking (soaked soybeans ‘S’), boiling (boiled soybeans ‘B’), and fermentation (fermented soybeans ‘F’), was examined in this study. Biochemical indices for the processed soybeans from each manufacturing step and those digested fractions by simulated in vitro gastrointestinal digestion were also evaluated. The result showed a significant (*P* < 0.05) increase in the protein digestibility of B (48.71 ± 0.04%) and F (50.21 ± 0.45%) compared to that of S (20.58 ± 0.25%), accompanying the accumulation of small protein sub-fractions and essential amino acids. Besides, antioxidant activity indices of all digested fractions increased around two to fourfold at the end of the simulated digestion. F showed a consistently increasing trend when the digestion stage progressed and maximum values overall at the final digestion stage.  Soybeans from fermentation step showed higher protein digestibility and indispensable amino acids as well as potential bioactivities than those from boiling and soaking step. The results demonstrated that manufacturing steps improved nutritional values of soybean protein, such as bioavailability of amino acids and certain bioactivities.

## Introduction

Although soybean has high nutritional value as a human diet, it contains several anti-nutritional factors such as trypsin and chymotrypsin inhibitors, which reduce protein digestion and bioavailability, and lectins that reduce nutrient absorption in the intestine^1^. These factors are, however, easily inactivated by dry heat treatment (roasting) or wet heat treatment (steaming or boiling)^1^. Thus, roasted or boiled soybeans are normally regarded as an edible form. Besides, fermentation has regionally and experientially been known as one of the manufacturing processes to be easily digestible soybeans.

Natto, a fermented soybean, is one of the Japanese traditional plant-based fermented foods using pure cultured *Bacillus subtilis* subsp. *natto*. It is well known that natto quality mainly depends on the fermentation conditions like temperature range and duration of the fermentation process. Meanwhile, soaking and boiling processes, which are the essential steps for preparing the fermentation process, are also recognized as the important processes for natto manufacturing and influence comprehensive natto quality, including nutritional perspectives. In fact, natto is comprised of fundamental nutrients, i.e., dietary fiber, isoflavones, linoleic acid, vitamins, and some minerals as well as some bioactive substances namely nattokinase (a fibrinolytic agent), gamma-polyglutamic acid (γ-PGA), and bioactive peptides^2,3^. The beneficial attributes of natto might be concerned with enzymatically degraded polymeric substances from soluble solids, particularly soluble nitrogenous compounds from fermentation by *B. subtilis* proteases which hydrolyze soy protein into oligopeptides and short-chain peptides during fermentation^4^.

Apart from the aforementioned factors, the digestive system significantly alters the release of new active fragments that present higher or lower bioactivity than raw materials after consumption. For example, protein availability at intestinal absorption can be estimated from protein digestibility, reflecting the efficiency of utilization of dietary protein^5^. The more peptide bonds are hydrolyzed, the lower molecular weight (MW) of oligopeptides and the more free amino acids are produced^6^. The amino acid profile is another vital factor in evaluating the protein nutritive quality. The digestion of that protein into small peptides and free amino acids is the principal factor for absorbing its amino acids by the human body^7^. For the soybean proteins such as glycinin and β-conglycinin, as a result of its hydrolysis during fermentation or digestion, bioactive peptides could be produced. They may exhibit bioactive properties and act like regulatory compounds, for instance, antioxidant, anti-diabetic, anti-hypertensive, antimicrobial, anti-inflammatory, and prevention of cancer and gastrointestinal disorders^8^. The natto consumption also contributes to improving the intestinal flora on both the composition and the metabolites aspect^9^. It was described that the odor of the faces was slightly reduced when consumed natto regularly, which related to protein digestion and metabolic activity of the human fecal flora^9^.

Nowadays, information on the health benefit of natto products is extensive. However, the information on changes in biochemical characteristics and potential health benefits of soybeans during the basic natto manufacturing steps, such as soaking, boiling, and fermentation, is limited. In other words, it is essential to verify whether the protein becomes easier digestible and is improved its biochemical properties during the manufacturing step. The purpose of this study was to investigate the effect of the manufacturing steps for fermented soybeans, i.e., soaking (as uncooked), boiling (as cooked), and fermentation, on the protein digestibility using a simulated in vitro gastrointestinal digestion technique. Digested fractions from those steps by simulated digestion were also investigated to evaluate potential health benefits such as in vitro antioxidant and anti-inflammatory activities.

## Results and discussion

### Digestive parameters for protein in vitro

**Figure 1:**
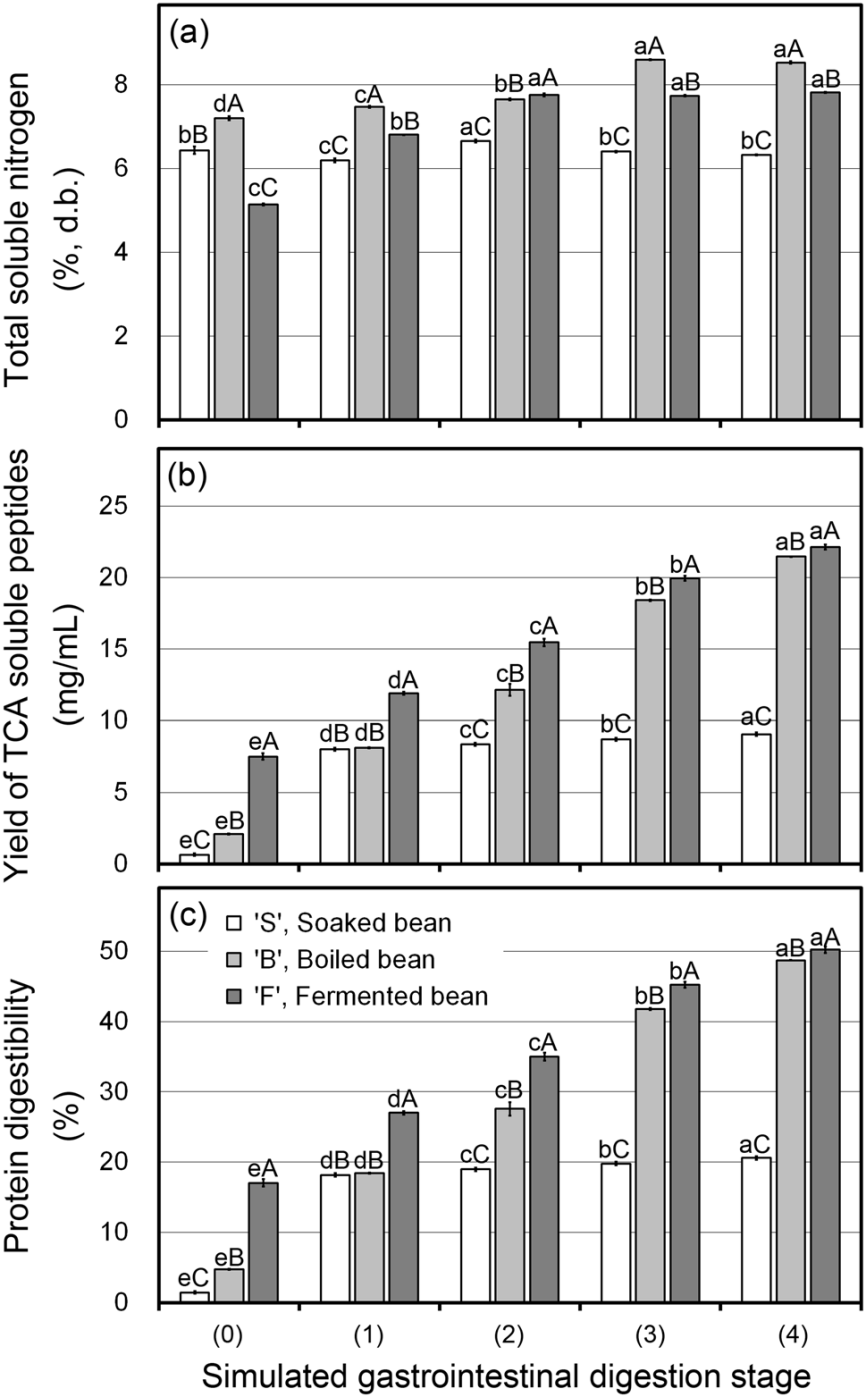
Change in total soluble nitrogen content (TSN) (**a**), yield of TCA-soluble peptides (**b**), and protein digestibility (**c**) of soaked (S), boiled (B), and fermented (F) soybeans at each digestion stage (0–4). Bars represent the standard deviation. Different lowercase and uppercase letters indicate significant differences (*P* < 0.05) among digestion stages and among manufacturing steps, respectively.

Figure 1 displays the variability in total soluble nitrogen (TSN) (%) (a), yield of trichloroacetic acid (TCA)-soluble peptides (mg/mL) (b) and protein digestibility (%) (c) during simulated digestion. The soybean samples are symbolized by the manufacturing steps, such as S (soaked), B (boiled), and F (fermented). The numbers shown in the x axis indicate an abbreviation for the simulated digestion stages; before digestion (stage 0), gastric digestion 1 h (stage 1), gastric digestion 2 h (stage 2), intestinal digestion 1 h after gastric digestion (stage 3) and intestinal digestion 2 h after gastric digestion (stage 4), respectively. The digested fraction of B showed the most TSN content followed by that in F and S as shown in Fig. 1a. TSN content during simulated digestion slightly increased in F and B, and mostly unchanged in S. In other words, nitrogen content rose approximately 1.20-fold between before and after simulated digestion in F and B. The increase of nitrogen content proved that more nitrogen was released during simulated digestion. However, not only nitrogen content but also digestible material should be examined to confirm the protein digestibility because TSN cannot represent amino acids and very short peptides. Thus, quantitative measurement of the short-chain peptides produced during simulated digestion performed using TCA-soluble peptides was determined to assess the protein digestibility.

Figure 1b displays changes in the yield of TCA-soluble peptide content during simulated digestion. It was shown that the progress of the digestion stage increased TCA-soluble peptides. The digested fraction of F showed the highest concentration of TCA-soluble peptides followed by that in B and S through the simulated digestion. The higher TCA-soluble peptide concentration before simulated digestion (stage 0) in F is due to the large amount of soluble peptides initially present generated by *B. subtilis* fermentation^10^. Then, during digestion, more TCA-soluble peptides were generated because of the reaction of digestive enzymes. Obviously, the yield of TCA-soluble peptides at the end of the simulated digestion (stage 4) for the soybeans collected at each manufacturing step was significantly higher than that of before digestion (stage 0) (*P* < 0.05). The smaller change of TCA-soluble peptides between before and after digestion in S could be understood by the presence of heat-sensitive protease inhibitors, especially trypsin inhibitor, which inhibits pancreatic protease, proteolysis, and protein absorption at the intestine^11,12^. Meanwhile, increases in TCA-soluble peptides during simulated digestion in B and F indicated that the protein digestibility was significantly improved compared to S.

Protein digestibility calculated from TCA-soluble peptides is shown in Fig. 1c. Protein digestibility increased as the digestion stage progressed. F showed the highest protein digestibility followed by B and S after the digestion. Compared to B and F, S showed stable and lower protein digestibility from digestion stages 1 to 4. The increase in protein digestibility in B and F, therefore, considered the digestive enzymes had digested the proteins that existed in the tissues and cells of the soybean in contrast to S, which possibly presented protease inhibitors. This indicates boiling and fermentation contribute to improving the protein digestibility of soybean. Meanwhile, the slight difference between F and B might concern the physical and/or biochemical changes in soybean by fermentation that could connect to the bioaccessibility of digestive enzymes^13‒15^.

From the other perspective, some nutritional compositions, such as crude protein, carbohydrate, fiber, ash, etc., can be associated with food digestive property. In addition, fundamental soybean nutritional compositions could be changed during fermentation^33^. Therefore, it should also be investigated that the impact of basic nutritional compositions of soybean and changes in protein components during digestion on the overall protein digestibility.

### Soluble protein fractions and distribution by sodium dodecyl sulfate polyacrylamide gel electrophoresis (SDS-PAGE) profile

**Figure 2:**
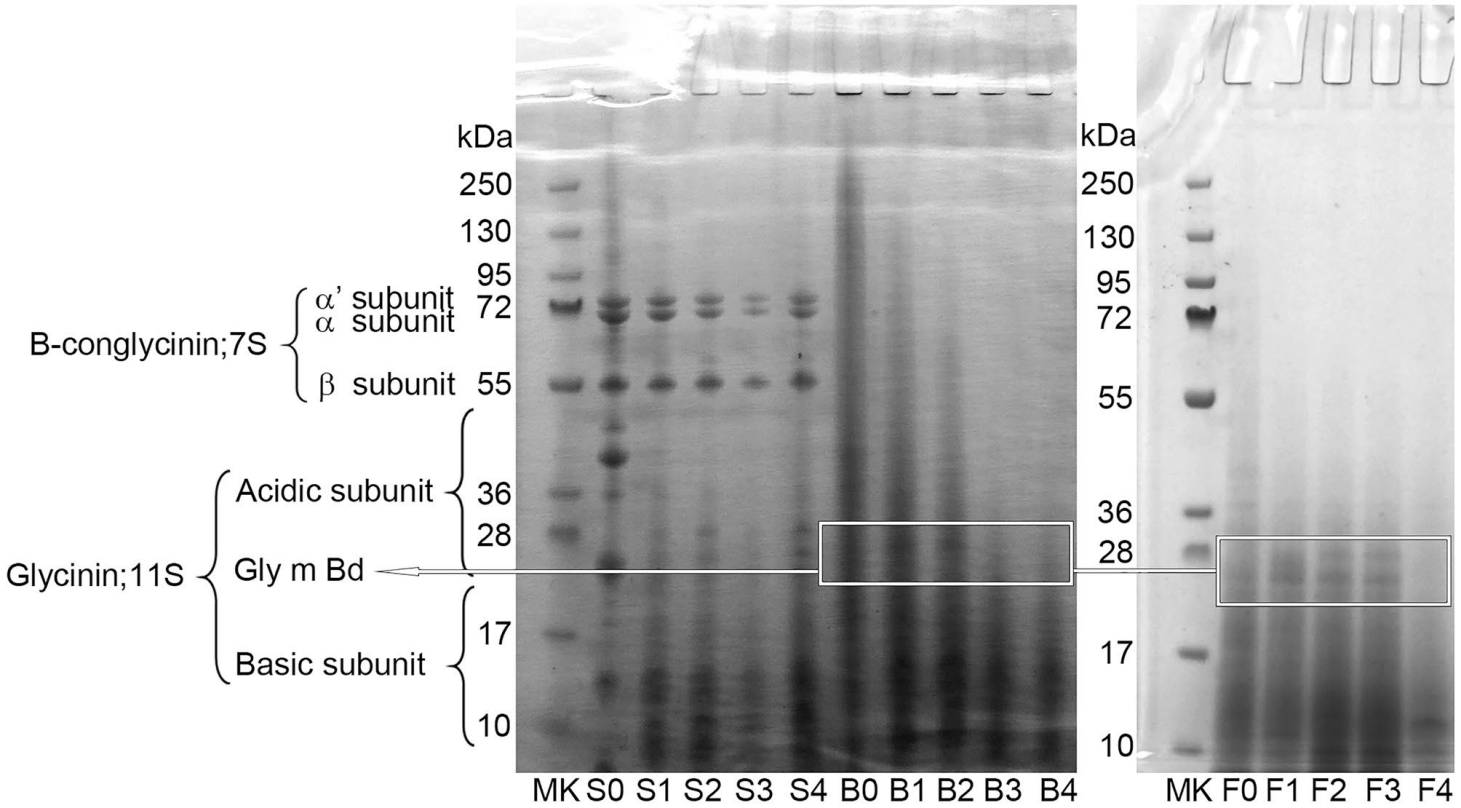
Changes in SDS-PAGE electrophoretogram for soaked (S), boiled (B), and fermented (F) soybean protein at each digestion stage. Lanes 1 and 12 represent a standard molecular marker (MK); lanes 2–6 present soaked bean before digestion (S0), gastric digestion 1 h (S1), gastric digestion 2 h (S2), intestinal digestion 1 h after gastric digestion (S3) and intestinal digestion 2 h after gastric digestion (S4), respectively; lanes 7–11 are for boiled bean in the same simulated digestion (B0–B4); lanes 13–17 are for fermented bean in the same simulated digestion (F0–F4).

The soluble protein distribution profile at each stage of simulated digestion for S, B and, F is shown in Fig. 2 (Each digestion stage is shown at the bottom and symbolized as S0–S4, B0–B4, F0–F4, respectively). Compared with the simply soaked soybean, S0, most proteins in B0 were broken down and hydrolyzed into lower MW peptides due to boiling. Besides, the proteins > 36 kDa were mostly eliminated by fermentation, resulting in an accumulation of low MW compounds as shown in F0. Apart from the microbial enzymes' action as fermentation, Fig. 2 also shows a change in the MW distribution of proteins during simulated digestion. For the soaked soybeans (S0–S4), the intensity of the protein bands corresponding to 7S (55–72 kDa) had remained but 11S (10–55 kDa) were looked like gradually diffused during simulated digestion. It could consider that the proteins > 55 kDa were hydrolyzed to lower MW peptides. In contrast, the high MW proteins for the boiled soybeans (B0–B4) were obviously disappeared by the progress of the simulated digestion. This should indicate the accumulation of small proteins was increased. For the fermented soybeans, it was observed that the MW distribution was stable though final stage F4 showed the ratio of the small protein fraction could be the highest. This also suggested that the abundance of oligopeptides with MW < 10 kDa had been increasing in F4. From the results, peptides with low MW were formed according to the degradation of some high MW peptides during simulated digestion. This suggests that digestive enzymes containing active proteases decompose the larger proteins. In pepsin hydrolysis at the gastric digestion stage, the subunits of fermented soybean protein were partially digested (F1 and F2), whilst in the intestinal digestion stage (F4), the most disappearances of larger molecules were found. This study verified that proteins were mainly digested to smaller MW size fragments that could be a prime contributing factor to superior bioavailability and benefit to human health. It can be concluded that cooking improves digestibility because it reduces anti-nutritional factors such as protease inhibitors (trypsin/chymotrypsin inhibitors), and fermentation makes more proteins degraded due to microbial enzymatic activity^16‒18^. Those compositions need to be further investigated in the future.

### Total phenolic content (TPC)

**Figure 3:**
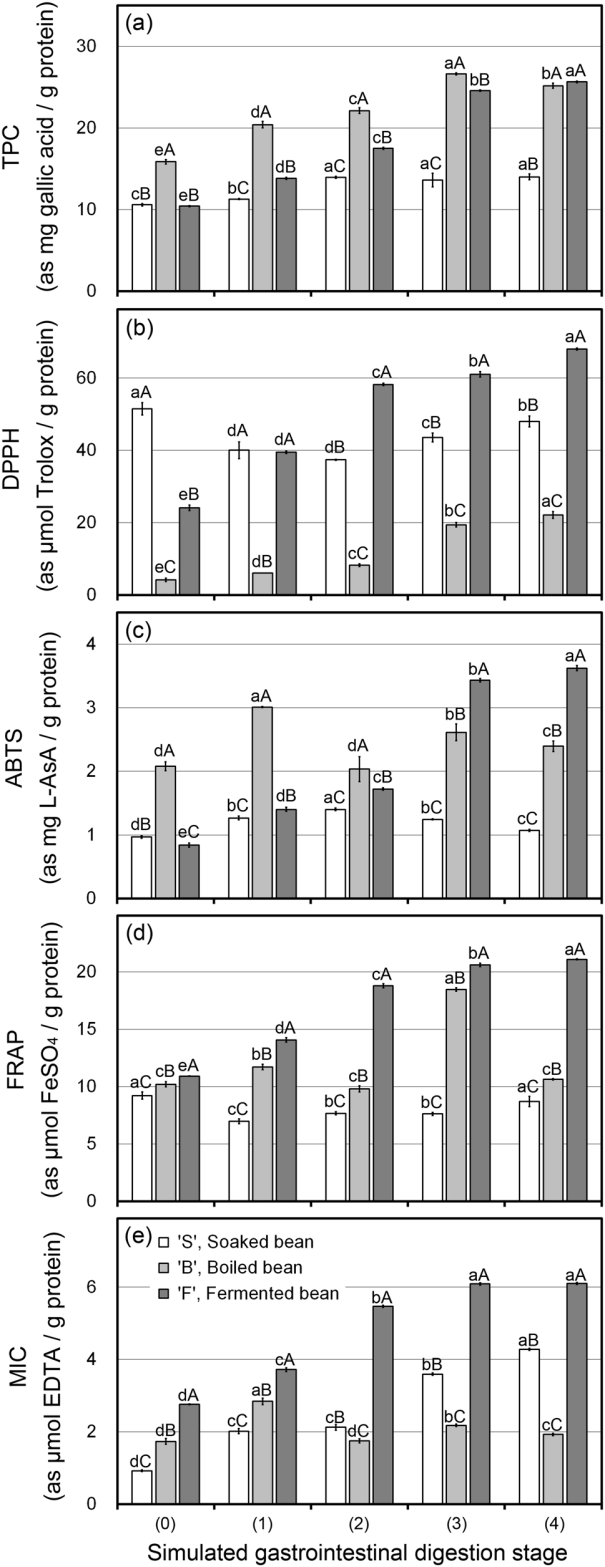
Changes in total phenolic content (TPC) (**a**), 2,2-diphenyl-1-picrylhydrazyl, 2, 2′ radical scavenging activity (DPPH) (**b**), 2-2′azinobis-(3-ethylbenzothiazoline-6-sulfonic acid) radical scavenging activities (ABTS) (**c**), ferric reducing antioxidant power (FRAP) (**d**), and chelating metal ion activity (MIC) (**e**) of soaked (S), boiled (B), and fermented (F) soybeans at each digestion stage. Bars represent the standard deviation from triplicate determinations. Different lowercase and uppercase letters indicate significant differences (*P* < 0.05) among digestion stages and among manufacturing steps, respectively.

Changes in TPC at each digestion stage of S, B, and F are shown in Fig. 3a. Overall, TPC escalated with the digestion stage progressed. A remarkable increment was observed through the digested fractions of B and F at each digestion stage. Meanwhile, it had comparatively been unchanged in S. Previous study^19^ described that digestive enzymes played a crucial role in exposing water-soluble polyphenols from the structure. Thus, the increases shown in B and F should be because of released soluble phenolics due to boiling and fermentation that might break tissue structures and/or cell matrices. Whereas, the increasing trend of F at the gastric stages 1 and 2 was different from B even though F was simply added the fermentation process to B. In this regard, it might concern the differences in the bioaccessibility between B and F, such as a slime-coated appearance of F, which could hamper or delay the penetration of digestive enzymes^13^. The presence of slime or mucilage could form sticky solutions or gels and impact passage rate, stickiness, and interactions with digestive enzymes and buffer solution in the stomach and small intestine^14,15^. It was also reported that an escalation in free phenolics is related to their antioxidant potential and expected to improve their bioavailability in the intestine^20^.

### Antioxidant activity

Changes in antioxidant activity during simulated digestion were evaluated by several indices such as scavenging activity on 2,2-diphenyl-1-picrylhydrazyl (DPPH), 2, 2′-azinobis-(3-ethylbenzothiazoline-6-sulfonic acid) (ABTS) radical scavenging activities, ferric reducing antioxidant power (FRAP) and chelating metal ion activity (MIC). The results are depicted in Fig. 3b (DPPH), Fig. 3c (ABTS), Fig. 3d (FRAP), and Fig. 3e (MIC). The initial values (stage 0) for all indices differed by the processes. In the progress of simulated digestion, the antioxidant activity of S except by MIC was mostly stable. In contrast, B showed a variable trend during simulated digestion. Meanwhile, F showed an increasing trend for all indices during simulated digestion. According to a previous study^21^, a reduction in DPPH was observed in two of the studied cultivars (EC4216 and BL2) of cowpea seeds after thermal treatment. It also described that phenolic compounds, especially tannins, were likely to form insoluble complexes with the cowpea seed protein under some thermal conditions^21^. This could explain the reduction of DPPH in B. However, F showed higher DPPH than B, although it was boiled before fermentation. It may be because the activity of F comes not only from phenolic compound reactions but also both phenolic compounds and oligopeptides^4^. Higher molecular weight phenolic compounds were depolymerized to simple phenolic monomers like catechins by metabolic activity of microbes^8,22^. Fermentation can also enhance the level of bioactive compounds by further breaking down cell walls of soybean seed leading to liberation of various bioactive compounds^8,22^. The same increasing trend during simulated digestion in F was observed for FRAP (Fig. 3d). The augmentation in FRAP of digests shows that proteins in F can be more effective in donating electrons after simulated digestion. The chelation of transition metals, namely Fe^2+^ and Cu^2+^, helps delay peroxidation and subsequently prevent food rancidity^23^. MIC in F also increased like DPPH and FRAP; however, MIC in B was mostly stable even though the phenolic compound content was higher (Fig. 3a).

It was reported that soybeans could present significant values of phenolic compounds such as chlorogenic, gallic, and protocatechuic acid^24^. The metal-binding potency of phenolic compounds is dependent upon the number and location of hydroxyl groups, properly oriented functional groups, and their unique phenolic structure, as well as the presence of ortho-dihydroxy polyphenols^25^. Even if a sample containing high polyphenols content, it might not show metal ion chelation activity because phenolic compounds can no longer bind metals once conjugated with a carbohydrate moiety, as in naturally occurring phenolic glycosides^22^. Apart from phenolic compounds, antioxidant activity changes during simulated digestion, suggesting that generated bioactive peptides might play the main role. In the digestion, pepsin possibly disrupted the spatial structure of soybean peptides conducive to binding and trapping metal ions and free radicals, resulting in reduced chelation and free-radical scavenging activity. In combination with pancreatic digestion, fully exposed or newly formed high-affinity metal-binding groups including imidazole and carboxylic groups, ionic and electrostatic interactions with metal ions were likely imposed^26^.

This study showed that antioxidant activity increased (in F) and was stable with partly (in B) or slight (in S) variations as the digestion stage progressed. The results distinctly stipulate that F in the last stage of simulated digestion showed the highest activity. This may be due to short-chain peptides convert free radicals into more stable products by donating electron atoms to cease the radical chain reaction^27^. From the results of higher antioxidant activity, peptides in F were assumed to resist the digestion and stay in the form with activity. Previous study^27^ described that factors including enzyme specificity, MW distribution, amino acid compositions, and the specific sequences of the peptides released largely affect the antioxidant properties of peptides. It was also reported that peptides with a lower MW (5–16 amino acids) have a higher probability of crossing the intestinal epithelium and exert better biological activity^28^. Figure 2 shows the smaller peptides found in SDS-PAGE, which may boost up antioxidant activity. The final digest stage of F (stage 4) showed the disappearance of larger MW peptides. It exhibited the highest overall antioxidant capacity. This study, thus, proved that fermented soybeans showed an enhancement of antioxidant activity with digestion progress and that generating antioxidant peptides tolerate digestive enzymes. This also suggested that active peptides reached the target site and showed an active reaction.

### Free amino acid composition

The change of free amino acid content between stage 0 and 4 was presented in Table 1. Free amino acids are expressed by the quantity (nmol/mL) of each individual amino acid. Based on the free amino acid content in S at stage 0, the total increment in S, B, and F at stage 4 was 2.15-, 6.78-, and 21.10-fold, respectively. The distinctive amino acids found in the soybeans were Ile, Glu, Val, Leu, Tyr, Phe, Lys, and Asg (> 20 nmol/mL). All amino acid contents increased by manufacturing steps and digestion; in particular, stage 4 in F showed the largest increment (Table 1). When comparing stages 0 and 4 in F, most amino acids were increased significantly, around 1.5- to 3.5-fold. For B, there was a notable difference in several amino acids between stages 0 and 4, such as a drastic increase in Leu, Tyr, Phe, Lys, and Arg, around 5- to 28-fold.

**Table 1:** Changes in free amino acid content in soaked, boiled, and fermented soybeans by simulated digestion.

Amino acids	‘S’, Soaked bean		‘B’, Boiled bean		‘F’, Fermented bean	
	Stage 0	Stage 4	Stage 0	Stage 4	Stage 0	Stage 4
Thr	0.58 ± 0.05	0.73 ± 0.05	0.00 ± 0.00	2.49 ± 0.04	1.93 ± 0.02	4.65 ± 0.06
Ser	0.87 ± 0.05	1.10 ± 0.05	0.00 ± 0.00	2.94 ± 0.04	3.13 ± 0.03	6.65 ± 0.06
Asp	1.12 ± 0.09	0.89 ± 0.00	0.00 ± 0.00	3.90 ± 0.01	3.45 ± 0.03	8.01 ± 0.73
Glu	4.37 ± 0.07	3.16 ± 0.01	0.00 ± 0.00	4.33 ± 0.03	26.66 ± 0.26	44.38 ± 0.24
Gly	0.58 ± 0.08	1.18 ± 0.07	0.00 ± 0.00	2.41 ± 0.06	5.17 ± 0.46	8.83 ± 0.95
Ala	3.35 ± 0.03	2.29 ± 0.13	11.91 ± 0.95	11.00 ± 0.85	6.77 ± 0.68	12.35 ± 0.34
Val	5.58 ± 0.02	5.69 ± 0.09	5.09 ± 0.36	9.65 ± 0.55	14.57 ± 0.67	26.70 ± 0.27
Met	0.62 ± 0.02	1.05 ± 0.02	0.00 ± 0.00	2.79 ± 0.06	6.46 ± 0.74	15.05 ± 0.61
Cysta*	1.96 ± 0.03	3.01 ± 0.06	1.98 ± 0.14	3.36 ± 0.09	4.78 ± 0.08	8.92 ± 0.54
Ile	0.57 ± 0.01	1.74 ± 0.02	0.00 ± 0.00	3.91 ± 0.07	9.18 ± 0.90	20.93 ± 0.31
Leu	0.63 ± 0.06	14.22 ± 0.26	0.00 ± 0.00	28.21 ± 0.45	24.98 ± 0.79	91.09 ± 0.82
Tyr	1.03 ± 0.07	8.01 ± 0.09	1.51 ± 0.14	25.68 ± 0.43	22.22 ± 0.23	86.40 ± 0.67
Phe	0.99 ± 0.05	15.58 ± 0.01	2.49 ± 0.20	41.73 ± 0.27	37.45 ± 0.53	123.78 ± 0.36
His	1.75 ± 0.12	1.65 ± 0.01	1.91 ± 0.03	4.07 ± 0.80	11.87 ± 0.36	18.87 ± 0.99
Lys	1.84 ± 0.13	3.81 ± 0.03	1.93 ± 0.05	18.48 ± 0.40	34.04 ± 0.35	133.58 ± 0.57
Trp*	n/a	n/a	n/a	n/a	n/a	n/a
Arg	10.80 ± 0.01	14.61 ± 0.08	14.90 ± 1.10	83.16 ± 0.14	3.23 ± 0.20	160.19 ± 0.99
Pro	0.00 ± 0.00	0.00 ± 0.00	0.00 ± 0.00	0.00 ± 0.00	2.56 ± 0.00	2.46 ± 0.00
Total	36.62 ± 0.89	78.73 ± 1.96	41.72 ± 2.97	248.11 ± 4.29	218.45 ± 6.33	772.86 ± 8.47

Each value represents the mean of three replications ± standard deviation.

*Cysteine (Cys) was determined in the form of cystathionine (Cysta). Tryptophan (Trp) cannot be reported because it is unstable and produces ammonia, so it was not obtained in the amino acid standard solution.

Table 2 shows the increment of amino acid contents in specific groups by the manufacturing steps and simulated digestion. The increment for each group was calculated as a mean of increment values of each amino acid categorized in each specific group. For “Increment (fold), compared to stage 0 in ‘S’", it was calculated as a mean of individual amino acid increment in each specific group by which dividing the quantity (nmol/mL) of each amino acid in B or F at stage 4 by that of in S at stage 0. For "Increment (fold), between stages 0 and stage 4", the increment of each amino acid was calculated by dividing the quantity of each amino acid in B or F at stage 4 by that of itself at stage 0. The values are expressed as fold-changes of increment. From the results, the manufacturing steps and digestion stage influenced the increment of amino acid content in all groups. Peptide bonds between HAA and AAA such as Phe, Trp, and Try are most effectively cleaved by pepsin. Pancreatin also acts including trypsin (cleaved peptide bonds at Arg and Lys sites), chymotrypsin (cleaved peptide bonds at Phe, Trp, Tyr, and Leu sites), and elastase (cleaved peptide bonds at Ala and other aliphatic amino acids)^27^. The results for amino acid content in this study show the same trend with Glu, Val, Tyr, Leu and, Phe in soybean meal fermented with *B. subtilis*^29^. The free amino acid composition formed upon digestion is widely referred to as the antioxidant peptide^30,31^. The notable increase in antioxidant properties shown in Fig. 3 could also be related to the escalation in free amino acid content. Thus, it was inferred that the digested fractions of processed soybeans in F and B, which showed an increase of amino acids by simulated digestion, could increase nutritional values and potentiate humans' physiological functions.

**Table 2:** Increment of amino acid contents in specific groups by the manufacturing steps and simulated digestion stages.

Amino acid group*	Increment (fold) compared to stage 0 in ‘S’		Increment (fold) between stages 0 and stage 4	
	Stage 4 in ‘B’	Stage 4 in ‘F’	‘B’	‘F’
EAA	112.29 ± 13.23	269.61 ± 27.83	9.52 ± 1.34	4.13 ± 0.47
HAA	97.40 ± 11.15	218.76 ± 25.04	5.29 ± 0.78	3.01 ± 0.33
AAA	45.22 ± 2.79	35.72 ± 5.27	13.04 ± 1.43	3.20 ± 0.35
AXA	32.29 ± 1.41	8.91 ± 1.51	6.93 ± 0.99	2.99 ± 0.32

*EAA = essential amino acids: Arg, His, Ile, Leu, Lys, Met, Phe, Thr, Try and Val; HAA = hydrophobic amino acids: Ala, Val, Ile, Leu, Tyr, Phe, Trp, Pro, Met and Cys; AAA = aromatic amino acids: Phe, Trp, Tyr and His; AXA = antioxidant amino acids: Trp, Tyr, Met, Cys, His, Phe and Pro.

### In vitro anti-inflammatory activity

**Figure 4:**
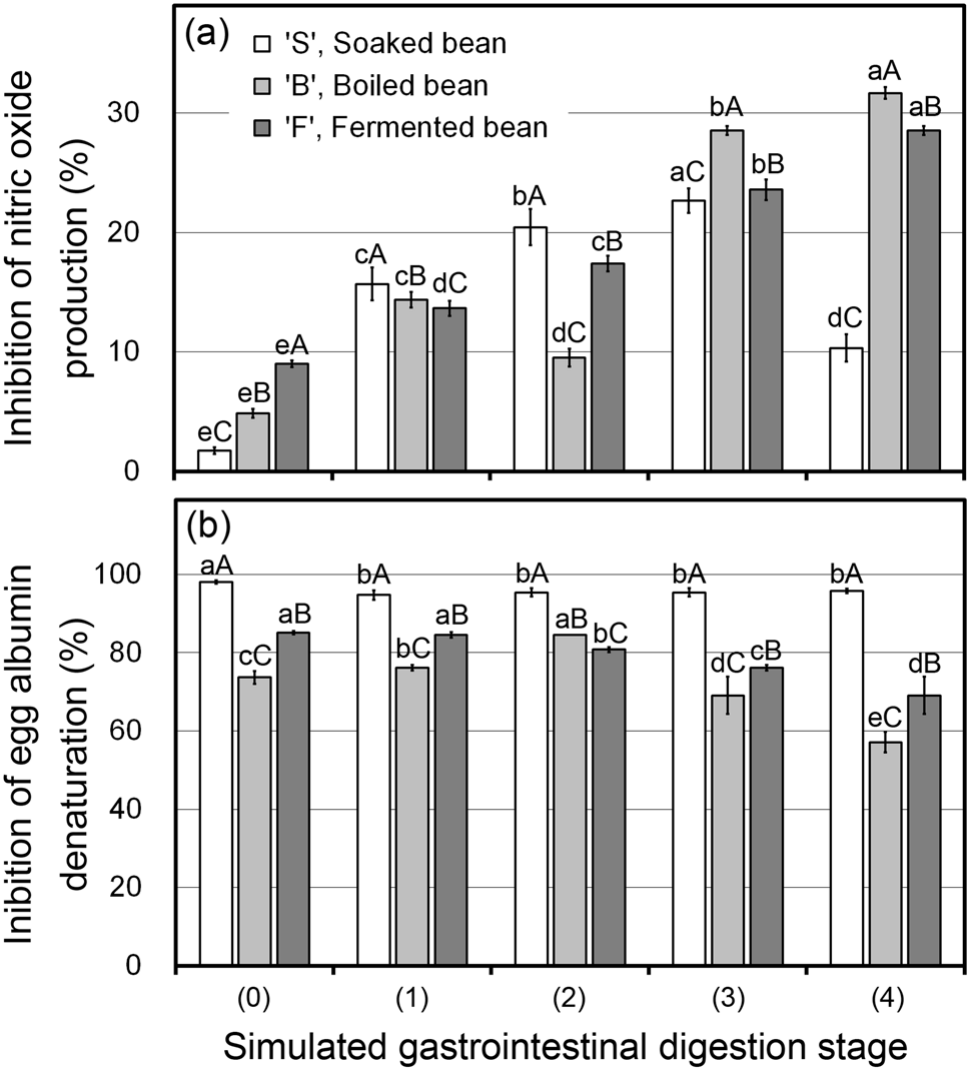
Changes in anti-inflammatory activity by the inhibition (%) of nitric oxide (NO) production (**a**) and egg albumin protein denaturation (**b**) of soaked (S), boiled (B), and fermented (F) soybeans at each digestion stage. Different lowercase and uppercase letters indicate significant differences (*P* < 0.05) among digestion stages and among manufacturing steps, respectively.

Figure 4 depicts variations on the anti-inflammatory activity of digested fractions for each manufacturing step tested using the inhibitions of NO production (a) and egg albumin denaturation (b). For the NO production inhibition (Fig. 4a), the inhibition (%) in F increased with the progress of the digestion stage. The inhibition (%) in B and S also showed a trend of increase basically, but B was unstable under the gastric stage (stages 1 and 2), and S showed a decrease between stages 3 and 4. Thus, the potential bioactivity against inflammation for peptides released from fermented soybeans during simulated digestion is notably assumed. Meanwhile, the value on the inhibition of egg albumin denaturation in F was comparatively stable than the result using NO production inhibition (Fig. 4b). By this method, digested fractions of S were the highest and stable. This result suggested that digested peptides could stabilize and were tolerant through the simulated digestion. The other factors which can play a role in anti-inflammatory action need to be considered, such as saponins, isoflavones, lignans, etc., presented in soybean, as well. They could show a strong anti-inflammatory effect and display a wide range of other bioactivities, including antioxidative, anti-cancer, anti-viral, cardiovascular protective effects, and hepatoprotective actions^8^. These interactive and complex effects should be examined in further study.

## Conclusions

Soaked soybean (S) showed the lowest TPC, protein digestibility with stability in antioxidant activity but higher egg albumin degeneration inhibition (%) with the digestion stage progressed. Boiling significantly affected TPC and antioxidant activity; thus, boiled soybean (B) showed higher values on them. Fermented soybean (F) showed the maximum antioxidant activity at the end of the simulated digestion (stage 4) that could be attributed to the synergistic combinations of several types of factors. From this perspective, boiling and fermentation improved overall protein digestibility and antioxidant properties after the digestion, possibly due to leaching out of bioactive compounds. It was also considered that the intense bioactivity in fermented soybean could be generated from the combination of the concentration of peptides/free amino acids, low MW peptides, and high percentages of antioxidative amino acid residues. The impact of basic nutritional compositions of soybean on the overall protein digestibility and the contribution of bioactivity compounds to the overall bioactivity and their profile should also be investigated.

## Methods

### Materials and reagents

Soybeans (*Glycine max* cv. Enrei) at the commercial maturity stage were purchased from the market in Ibaraki prefecture, Japan on January 2019, and kept in a refrigerator (4 °C) before the experiment. Digestive enzymes for gastric digestion used porcine gastric mucosa pepsin (800–2500 U/mg protein; EC 3.4.23.1) and for intestinal digestion used porcine pancreas pancreatin (8 × USP specifications; EC 232-468-9,), invertase from baker’s yeast (grade VII, ≥ 300/mg solid) and amyloglucosidase (3260 U/mL), They were procured from Sigma-Aldrich Chemical Co. (St. Louis, MO, USA) and Megazyme International Ireland Ltd (Wicklow, Ireland). All other chemicals of analytical grade were purchased from Wako Pure Chemical Corporation (Tokyo, Japan).

### Statement for soybean samples

The collection of soybeans complies with relevant institutional, national, and international guidelines and legislation. The sample soybeans were certified the quality and permission of the usage by the Japanese Agricultural Standards (JAS), which was established by the Minister of Agriculture, Forestry and Fisheries of Japan.

### Sample preparation

The dehulled yellow-seeded soybean samples (approx. 900 g) were washed using tap water and soaked in distilled water (soybeans/water ratio of 1 : 3, w/v) for 18 h at 20 °C. Then, the soaked samples were separated from the soaking water. At this step, a part of the soaked samples (approx. 300 g) was collected as soaked soybeans, ‘S’. Subsequently, soaked samples were washed again with tap water, boiled with the same ratio of fresh distilled water using a household pressure cooker (H-5040, Pearl Metal Co., Ltd., Niigata, Japan) under approximately 100 kPa gauge pressure (approx. 120 °C) for 90 min, and then cooled down at room temperature. At this step, a part of the boiled samples (approx. 300 g) was collected as boiled soybeans, ‘B’. Fermented soybeans were also prepared from the freshly prepared boiled soybeans. The boiled soybeans (approx. 150 g) were transferred into a sterilized glass beaker and inoculated with 50 mL of the diluted culture of *Bacillus* spp. *natto* from a commercial natto product S-903 (Takanofoods Co., Ltd, Ibaraki, Japan). After inoculation, the inoculated soybeans (37.5 g) were packed into a paper cup (205 mL size), the top surface covered with polyvinylidene chloride wrap film, and incubated in an incubator (MIR-253; Sanyo, Osaka, Japan) at 40 °C for 18 h. The products were collected and considered as fermented soybeans, 'F'.

### Simulated in vitro gastrointestinal digestion

The simulated static in vitro gastrointestinal digestion model described previously^32^ was used with minor adjustment and carried out in duplicate. The soybean samples from each manufacturing step (15% protein, wet weight) (around 150 g) were blended with gastric buffer in a glass reactor and homogenized using a homogenizer (NS-52K, Physcotron, Microtec Co., Ltd, Chiba, Japan) at 10,000×*g* for 5 min before starting the experiment. The temperature of the reactor was maintained at 37 °C throughout the experiment, and the pH of the sample was adjusted to 1.20 with 3 M HCl before starting the gastric stage. The gastric stage was initiated by the addition of pepsin solution. After 2 h of the gastric stage, the pH of the sample was adjusted to 6.80 using 3 N NaOH to inactivate the pepsin. The small intestinal stage was initiated after finishing the gastric stage by addition of intestinal enzyme solution. The sample was maintained at the intestinal conditions for 1 and 2 h. The supernatant was separated, collected and lyophilized for use in further analysis. Sampling of 5 sample sets was performed separately from each digestion stage. The digestion stages means as follows: ‘stage 0’ for samples before digestion, ‘stage 1’ for samples from gastric digestion for 1 h, ‘stage 2’ for samples from gastric digestion for 2 h, ‘stage 3’ for samples after gastric digestion for 2 h and intestinal digestion for 1 h, and ‘stage 4’ for samples after gastric digestion for 2 h and intestinal digestion for 2 h (the end of the simulated digestion). The following analyses were carried out in triplicate.

### Determination of total soluble nitrogen and protein content

A CN coder (MT-700 Mark 2; Yanako, Tokyo, Japan) was employed to determine the total soluble nitrogen (TSN) content based on the Dumas principle. The hippuric acid (200-37032; Kishida Chemical, Osaka, Japan) was used as a standard to calibrate the measured values following the manufacturer’s instructions^32^. The amount of protein present in the sample was calculated by nitrogen content and then convert to protein using a conversion factor of 6.25.

### TCA-soluble peptide content

Quantitative measurement of the short-chain peptides performed using TCA-soluble peptides can be used to assess protein digestibility during simulated digestion^33^. TCA-soluble peptide content of the processed and digested soybean samples was analyzed following the method^34^ with minor modifications^32^. The content of TCA-soluble peptide content was calculated as micromoles of tyrosine per gram of sample^35^. Protein digestibility was expressed as the percentage of TCA-soluble peptide content in the supernatant of the sample during simulated digestion and compared to the total protein content of soaked soybeans. The protein digestibility was calculated using Eq. (1).

1 $${\text{Protein}}\;{\text{digestibility}}\;(\% ){\text{ }} = {\text{ B/A }} \times {\text{ }}100$$

where A is the total protein content of soaked soybeans, and B is the TCA-soluble peptide content at each digestion stage.

### Soluble protein fractions and distribution by electrophoretic analysis

Before studying protein patterns, Biuret method^36^ using a standard bovine serum albumin (BSA) was employed for determination of the protein content of supernatants collected at different stages of simulated digestion. Then, a sodium dodecyl sulfate polyacrylamide gel electrophoresis (SDS-PAGE) technique was applied to determine the protein patterns. The protein solution was diluted with deionized water to be the same concentration. The samples (all containing 20 μg of protein per well) and the protein standard markers (Thermo Scientific, Rockford, IL, USA) were loaded onto NuPAGE Bis–Tris gradient precast gel (4–12% gradient, 10 × 10 cm^2^) in a Novex XCell Mini-Cell (Invitrogen, Thermo Scientific, Rockford, IL, USA) following the previous method^32^.

### Free amino acid analysis

An automatic amino acid analyzer (JLC-500/V2 equipped with an ion exchange column; Jeol, Tokyo, Japan) was used for determination of free amino acid content following the previous study^32^. The increment of amino acids in the specific groups was calculated by; (1) dividing the quantity (nmol/mL) of each amino acid in soaked soybeans for stage 0 by the values of processed soybeans for stage 0 (compared to soaked bean); (2) dividing the values in the samples before (stage 0) by after digestion (stage 4) for each manufacturing step (between digestion). The values are expressed as fold-changes of increment.

### Biochemical properties

Total phenolic content (TPC) was determined and reported as the milligrams of gallic acid equivalent (GAE) per gram of protein (dry matter) following the method of previous study^32^.

For the determination of antioxidant activity, several parameters such as scavenging activity on 2,2-diphenyl-1-picrylhydrazyl (DPPH), 2, 2′-azinobis-(3-ethylbenzothiazoline-6-sulfonic acid) (ABTS) radical scavenging activities, ferric reducing antioxidant power (FRAP) and chelating metal ion activity (MIC) were applied following the method previously reported^32^. The DPPH was calculated using a Trolox standard curve (0–1000 μmol/L) and expressed as micromoles of Trolox equivalent (TE) per gram of protein (dry matter). The ABTS was expressed as milligrams of ascorbic acid equivalent (AA) per gram of protein (dry matter). The FRAP was calculated from an FeSO_4_ standard curve (0–100 μmol/L) and expressed as micromoles of FeSO_4_ equivalent per gram of protein (dry matter). The MIC was expressed as micromoles of EDTA equivalent per gram of protein (dry matter).

For the determination of in vitro anti-inflammatory activity, the inhibition of nitric oxide (NO) production and the inhibition of egg albumin denaturation were determined following the methods previously reported^37,38^, respectively. These inhibition (%) were calculated using Eq. (2).

2 $${\text{Inhibition}}\;~(\% ) = ~\left( {1 - \frac{{A_{{sample}} }}{{A_{{control}} }}} \right)~ \times 100$$

where A_control_ is the absorbance of distilled water as a control, and A_sample_ is the absorbance in the presence of the samples or standards. Gallic acid was used as a standard for the inhibition of NO production and diclofenac sodium was used as a standard for the inhibition of egg albumin denaturation.

### Experimental design

All sample preparation processes and the simulated in vitro gastrointestinal digestion were carried out in duplicate. Sampling from all manufacturing steps and each simulated digestion stage was carried out in triplicate.

### Statistical analysis

Statistical software (SPSS 26, IBM, Armonk, New York, USA) was used for Duncan’s multiple range test for mean comparisons and analysis of variance. Differences were considered significant at *P* < 0.05.

### Ethical approval

This article does not contain any studies with human participants or animals performed by any of the authors.

## Data Availability

The research data of this study will be provided upon request.

## References

[1] Liener IE (1994). Implications of antinutritional components in soybean foods. Crit. Rev. Food Sci. Nutr..

[2] Weng Y, Yao J, Sparks S, Wang KY (2017). Nattokinase: An oral antithrombotic agent for the prevention of cardiovascular disease. Int. J. Mol. Sci..

[3] Escamilla DM (2019). Improvement of soybean cultivars for natto production through the selection of seed morphological and physiological characteristics and seed compositions: A review. Plant Breed..

[4] Yang Y (2016). Secondary structure and subunit composition of soy protein in vitro digested by pepsin and its relation with digestibility. Biomed. Res. Int..

[5] González Montoya M, Hernández Ledesma B, Mora Escobedo R, Martínez Villaluenga C (2018). Bioactive peptides from germinated soybean with anti-diabetic potential by inhibition of dipeptidyl peptidase-IV, α-amylase, and α-glucosidase enzymes. Int. J. Mol. Sci..

[6] Weng TM, Chen MT (2010). Changes of protein in natto (a fermented soybean food) affected by fermenting time. Food Sci. Technol. Res..

[7] Chen CC, Shih YC, Chiou PWS, Yu B (2010). Evaluating nutritional quality of single stage- and two stage-fermented soybean meal. Asian-Australas. J. Anim. Sci..

[8] Sanjukta S, Rai AK (2016). Production of bioactive peptides during soybean fermentation and their potential health benefits. Trends Food Sci. Technol..

[9] Fujisawa T, Shinohara K, Kishimoto Y, Terada A (2006). Effect of miso soup containing Natto on the composition and metabolic activity of the human faecal flora. Microb. Ecol. Health Dis..

[10] Kiers JL, Van Laeken AEA, Rombouts FM, Nout MJR (2000). In vitro digestibility of <i>Bacillus</i> fermented soya bean. Int. J. Food Microbiol..

[11] Chi CH, Cho SJ (2016). Improvement of bioactivity of soybean meal by solid-state fermentation with *Bacillus**amyloliquefaciens* versus *Lactobacillus* spp. and *Saccharomyces**cerevisiae*. LWT Food Sci. Technol..

[12] Martinez-Gonzalez AI (2017). Polyphenolic compounds and digestive enzymes: In vitro non-covalent interactions. Molecules.

[13] Hu Y (2010). Characterization of fermented black soybean natto inoculated with *Bacillus natto* during fermentation. J. Sci. Food Agric..

[14] Xu K (2020). Okra seed and seedless pod: Comparative study of their phenolics and carbohydrate fractions and their impact on bread-making. Food Chem..

[15] Fiszman S, Varela P (2013). The role of gums in satiety/satiation. A review. Food Hydrocoll..

[16] Frias J (2008). Fermented soyabean products as hypoallergenic food. Proc. Nutr. Soc..

[17] Aguirre L, Garro MS, Savoy de Giori G (2008). Enzymatic hydrolysis of soybean protein using lactic acid bacteria. Food Chem..

[18] Wang T, Qin GX, Sun ZW, Zhao Y (2014). Advances of research on glycinin and β-conglycinin: A review of two major soybean allergenic proteins. Crit. Rev. Food Sci. Nutr..

[19] Zhang B (2020). A review on insoluble-bound phenolics in plant-based food matrix and their contribution to human health with future perspectives. Trends Food Sci. Technol..

[20] Bhanja T, Kumari A, Banerjee R (2009). Enrichment of phenolics and free radical scavenging property of wheat koji prepared with two filamentous fungi. Bioresour. Technol..

[21] Yadav N (2018). Effect of thermal and non-thermal processing on antioxidant potential of cowpea seeds. Int. J. Food Prop..

[22] Moktan B, Saha J, Sarkar PK (2008). Antioxidant activities of soybean as affected by *Bacillus*-fermentation to kinema. Food Res. Int..

[23] Zhang L, Li J, Zhou K (2010). Chelating and radical scavenging activities of soy protein hydrolysates prepared from microbial proteases and their effect on meat lipid peroxidation. Bioresour. Technol..

[24] Silva MO, Brigide P, de Toledo NMV, Canniatti-Brazaca SG (2018). Phenolic compounds and antioxidant activity of two bean cultivars (*Phaseolus**vulgaris* L.) submitted to cooking. Braz. J. Food Technol..

[25] Andjelković M (2006). Iron-chelation properties of phenolic acids bearing catechol and galloyl groups. Food Chem..

[26] Luzardo-Ocampo I (2017). Bioaccessibility and antioxidant activity of free phenolic compounds and oligosaccharides from corn (<i>Zea</i> <i>mays</i> L.) and common bean (<i>Phaseolus</i> <i>vulgaris</i> L.) chips during in vitro gastrointestinal digestion and simulated colonic fermentation. Food Res. Int..

[27] Aluko RE, Shahidi F (2015). Amino acids, peptides, and proteins as antioxidants for food preservation. Handbook of Antioxidants for Food Preservation.

[28] Ketnawa S, Martínez-Alvarez O, Benjakul S, Rawdkuen S (2016). Gelatin hydrolysates from farmed Giant catfish skin using alkaline proteases and its antioxidative function of simulated gastro-intestinal digestion. Food Chem..

[29] Song YS (2008). Immunoreactivity reduction of soybean meal by fermentation, effect on amino acid composition and antigenicity of commercial soy products. Food Chem..

[30] Ajibola CF, Fashakin JB, Fagbemi TN, Aluko RE (2011). Effect of peptide size on antioxidant properties of african yam bean seed (*Sphenostylis stenocarpa*) protein hydrolysate fractions. Int. J. Mol. Sci..

[31] Sánchez A, Vázquez A (2017). Bioactive peptides: A review. Food Qual. Saf..

[32] Ketnawa S, Ogawa Y (2019). Evaluation of protein digestibility of fermented soybeans and changes in biochemical characteristics of digested fractions. J. Funct. Foods.

[33] Shrestha AK, Dahal NR, Ndungutse V (2010). Bacillus fermentation of soybean: A review. J. Food Sci. Technol. Nepal.

[34] Chen N, Zhao M, Sun W (2013). Effect of protein oxidation on the in vitro digestibility of soy protein isolate. Food Chem..

[35] Wang J, Chi Y, Cheng Y, Zhao Y (2018). Physicochemical properties, in vitro digestibility and antioxidant activity of dry-heated egg white protein. Food Chem..

[36] Gornall AG, Bardawill CJ, David MM (1949). Determination of serum proteins by means of the biuret reaction. J. Biol. Chem..

[37] Harsha SN, Anilakumar KR, Mithila MV (2013). Antioxidant properties of *Lactuca sativa* leaf extract involved in the protection of biomolecules. Biomed. Prev. Nutr..

[38] Osman NI (2016). In vitro xanthine oxidase and albumin denaturation inhibition assay of <i>Barringtonia</i> <i>racemosa</i> L. and total phenolic content analysis for potential anti-inflammatory use in gouty arthritis. J. Intercult. Ethnopharmacol..

